# Chiropractic manipulation in pediatric health conditions – an updated systematic review

**DOI:** 10.1186/1746-1340-16-11

**Published:** 2008-09-12

**Authors:** Allan Gotlib, Ron Rupert

**Affiliations:** 1 Canadian Chiropractic Association, CMCC Homewood Professor, 30 St. Patrick St. Suite 600, Toronto, Ontario, M5T 3A3, Canada; 2 Parker College of Chiropractic, 2500 Walnut Hill Lane, Dallas, Texas 75229, USA

## Abstract

**Objective:**

Our purpose was to review the biomedical literature from January 2004 to June 2007 inclusive to determine the extent of new evidence related to the therapeutic application of manipulation for pediatric health conditions. This updates a previous systematic review published in 2005. No critical appraisal of the evidence is undertaken.

**Data Sources:**

We searched both the indexed and non-indexed biomedical manual therapy literature. This included PubMed, MANTIS, CINAHL, ICL, as well as reference tracking. Other resources included the Cochrane Library, CCOHTA, PEDro, WHO ICTRP, AMED, EMBASE and AHRQ databases, as well as research conferences and symposium proceedings.

**Results:**

The search identified 1275 citations of which 57 discrete citations met the eligibility criteria determined by three reviewers who then determined by consensus, each citation's appropriate level on the strength of evidence scale. The new evidence from the relevant time period was 1 systematic review, 1 RCT, 2 observational studies, 36 descriptive case studies and 17 conference abstracts. When this additional evidence is combined with the previous systematic review undertaken up to 2003, there are now in total, 2 systematic reviews, 10 RCT's, 3 observational studies, 177 descriptive studies, and 31 conference abstracts defining this body of knowledge.

**Summary:**

There has been no substantive shift in this body of knowledge during the past 3 1/2 years. The health claims made by chiropractors with respect to the application of manipulation as a health care intervention for pediatric health conditions continue to be supported by only low levels of scientific evidence. Chiropractors continue to treat a wide variety of pediatric health conditions. The evidence rests primarily with clinical experience, descriptive case studies and very few observational and experimental studies. The health interests of pediatric patients would be advanced if more rigorous scientific inquiry was undertaken to examine the value of manipulative therapy in the treatment of pediatric conditions.

## Introduction

This paper updates a previous systematic review published in 2005 which assessed the evidentiary basis for health intervention claims made by chiropractors with respect to pediatric patients and manipulation [1]. In addition, the review examined the range of pediatric health conditions in which chiropractors utilize manipulation as a health intervention.

In 2005 Gotlib and Rupert concluded that health claims made by practitioners with respect to the application of manipulation as a health care intervention for pediatric health conditions, were supported primarily by only low levels of scientific evidence. Chiropractors employ manipulation for the treatment of a wide variety of pediatric health conditions. The evidentiary basis rests primarily with clinical experience, descriptive case studies and a few RCT's. They identified the need for more rigorous scientific inquiry to examine the value of manipulative therapy in the treatment of pediatric conditions.

The objective of this update was to summarize the indexed and non-indexed biomedical literature up to and including June 2007 and to determine the extent of new evidence related to the therapeutic application of manipulation for pediatric health conditions. No critical appraisal of the evidence is undertaken.

In utilizing a health care intervention, health care providers should be guided by relevant systematic reviews and high quality randomized controlled clinical trials. This advances the health interests of patients. When systematic reviews and RCT's are absent, clinicians, including chiropractors, have relied upon lower levels of evidence, clinical observation and best practices when available.

In a more narrowed and specialized clinical milieu, in which patient-centered risks, benefits, effectiveness and safety remain undefined, health care providers must measure carefully, their claims of efficacy and safety. This would be consistent both with scientific evidence-based decision making and growing expectations of patients.

## Methods

### Pediatric data sources in biomedical literature

The search strategy set out in Table 1 captures the peer reviewed biomedical literature related to the manual therapy sector.

**Table 1:** Database sources

Source	Files Retrieved	Files Accepted
PubMed	82	5
ICL	63	27
CCOHTA	2	0
AHRQ	195	0
PEDro	20	0
WHO ICTRP	62	0
Cochrane	291	0
CINAHL	181	20
MANTIS	49	20
AMED	14	4
EMBASE	295	5
Conference Proceedings	20	17
Manual searching	1	1
		
	1275	99

We searched both the indexed and non-indexed manual therapy sector. This included PubMed, MANTIS, CINAHL, ICL, as well as reference tracking. Other resources included the Cochrane Library (includes the Cochrane Complementary Medicine Field Register of Controlled Trials), CCOHTA (CADTH), PEDro, WHO ICTRP (includes  which lists trials currently recruiting or completed but not yet reported), AMED, EMBASE and AHRQ databases.

We also searched conference and symposium proceedings of the World Federation of Chiropractic Congress (2005, 2007), Symposium of the Consortium of Canadian Chiropractic Research Centers (2004, 2007), Research Agenda Conference and the Association of Chiropractic Colleges (2004, 2005, 2006, 2007).

At the conclusion of these search procedures, all references were screened to preclude duplication.

### Definitions

The pediatric age range was defined as 0 – 18 years inclusive. Manipulation was defined as the application of a high velocity short amplitude thrust to a spinal or peripheral joint. Treatment was defined as the application of manipulation in a therapeutic clinical context.

### Levels of evidence

The Scale of Evidence in hierarchy employed by the three reviewers was systematic reviews, experimental studies (RCT), observational studies (quasi-experimental studies controlled but not randomized such as case-controlled studies or cohort studies), descriptive studies (non-controlled non-randomized studies such as case series, case reports, surveys, literature reviews, and expert opinion) and finally abstracts from conferences.

### Inclusion and exclusion criteria

The inclusion and exclusion criteria for documents are set out in Table 2. Three reviewers agreed on the eligible articles retrieved from the data sources set out in Table 1. Three reviewers agreed on the placement of each paper on the evidence scale. Those papers not meeting the inclusion criteria were deleted.

**Table 2:** Inclusion and exclusion criteria

***Inclusion criteria***
• study includes children age 0 – 18 inclusive; and
• study investigates manipulation in a therapeutic clinical context; and
• study was published in a peer reviewed publication, or was reported at Conference Proceedings; or
• relevant systematic review.

***Exclusion criteria***

• descriptive studies (ie surveys) that do not investigate manipulation in a therapeutic clinical context,
• studies in which a small number of pediatric subjects were a part of a larger adult trial, and the results are not reported separately,
• abstracts from conference proceedings which were later published in the scholarly literature.

## Results

We identified 1275 citations. Of these, 57 discrete documents met the eligibility criteria for the study (see Table 3) determined by three reviewers who then determined by consensus, each citation's appropriate level on the strength of evidence scale. The bibliography is available to readers on request.

**Table 3:** Level of evidence

Level of evidence		# of studies 2004–2007		# of studies up to 2003		total # studies to June 2007
systematic reviews		1		1		2
RCT's		1		9		10
Observational		2		1		3
Descriptive		36		141		177
Conference abstracts		17		14		31
RCT	0		5		5	
OBSERV	1		0		1	
DESC	16		9		25	

The new evidence from the relevant time period (January 2004 to June 2007) was 1 systematic review, 1 RCT, 2 observational studies, 36 descriptive case studies and 17 conference abstracts.

When this additional evidence is combined with the previous systematic review undertaken up to 2003, there are now in total, 2 systematic reviews, 10 RCT's, 3 observational studies, 177 descriptive studies, and 31 conference abstracts defining this body of knowledge (see Table 3).

### Systematic review

One systematic review on infantile colic was completed by CCOHTA in December 2003 [2]. Four reports met the review's inclusion criteria. These reports described four randomized controlled trials (two published in peer-reviewed journals, one conference abstract and one unpublished manuscript) with spinal manipulation in all trials performed by chiropractors. Quality scores were measured by the Jadad scale. The systematic review concluded that (1) there is no convincing evidence that spinal manipulation alone can affect the duration of infantile colic symptoms, (2) the effect of spinal manipulation on sleep time, parental anxiety, quality of life and the number of infants meeting diagnostic criteria for colic could not be determined using available evidence, and (3) the potential harm from the spinal manipulation of infants with colic could not be determined using evidence available from controlled trials.

### Systematic review 2007 update

One systematic review on the effects of manual therapy of kinetic imbalance due to suboccipital strain syndrome (KISS) in infants with positional preference, plagiocephaly and colic was reported in 2005 [3]. The systematic review concluded there is no scientific evidence that spinal manipulation is useful in infants with signs and symptoms of the proposed KISS syndrome.

### Randomized controlled trials

The 9 RCT's involved a total of 590 children [4-12]. Two trials on asthma involved 80 and 36 children respectively. All spinal manipulation was performed by chiropractors. The first trial concluded that in children with mild or moderate asthma, the addition of chiropractic spinal manipulation to usual medical care provided no benefit. The second trial concluded that after 3 months of combining chiropractic spinal manipulation with optimal medical management for pediatric asthma, the children rated their quality of life substantially higher and their asthma severity substantially lower.

Two trials on enuresis involved 171 and 46 children respectively. All spinal manipulation was performed by chiropractors. The first trial concluded the study results do not support the claim that chiropractic care in enuretic children is an effective therapy for this condition. The second trial concluded that the study results strongly suggest the effectiveness of chiropractic treatment for primary nocturnal enuresis.

Two trials on infantile colic involved 50 and 86 children respectively. All spinal manipulation was performed by a chiropractor. In the first trial the study concluded that spinal manipulation is effective in relieving infantile colic. In the second trial the study concluded that chiropractic manipulation is no more effective than placebo in the treatment of infantile colic.

One trial on chronic otitis media was a feasibility study involving 22 children with spinal manipulation performed by a chiropractor and concluded that recruitment for a randomized controlled trial is feasible and could be enhanced by medical collaboration.

One trial on jet lag involved 15 children with spinal manipulation performed by a chiropractor and concluded that chiropractic care did not reduce the effects of jet lag.

One trial on radial head subluxation involved 84 children. Manipulation of the radial head was performed by a physician. The study concluded that in the reduction of radial head subluxations, the hyperpronation technique required fewer attempts at reduction compared with supination, was successful more often than supination, and was often successful when supination failed.

### Randomized controlled trials 2007 update

One RCT on adolescent idiopathic scoliosis reported in 2006 was a pilot study involving 6 children with manipulation performed by chiropractors [13]. The investigators concluded that this pilot study showed the viability for a larger randomized trial.

### Observational studies

There was one observational study involving 24 children with manipulation performed by chiropractors [14]. It concluded that chiropractic treatment was effective for the wide range of symptoms associated with "learning and behavioural impairments resulting from brain damage and/or neurological dysfunction accompanied by impairing emotional overlay".

### Observational studies 2007 update

Two observational studies involved 49 children and all manipulation was performed by chiropractors [15,16]. One study reported in 2006, involved 14 children with autism, and compared upper cervical (Atlas Orthogonal technique) provided to 7 children with full spine manipulation provided to 7 children. The study concluded that the clinical improvement of autistic children under full spine chiropractic care was enhanced four fold when the technique of adjustment was shifted to upper cervical AO technique.

One study in 2004 examined 25 infants demonstrating difficulties with breastfeeding and compared 10 infants without complaint. The study concluded that soft tissue work, cranial therapy, and spinal adjustments may have a direct result in improving the infant's ability to suckle efficiently.

### Descriptive studies

The 141 descriptive studies involved a total of 2245 children. The literature retrieved reveals a host of conditions reported by a number of practitioners, primarily chiropractors, who claim to successfully treat a variety of pediatric health conditions with manipulation. The conditions include, but are not limited to the following: scoliosis, congenital torticollis, juvenile arthritis, strabismus, foot inversion, neurologic performance (learning, behaviour, attention), enuresis, Erb's palsy, infantile colic, asthma, esophoria, fever, shoulder impingement, encopresis, neurogenic bladder, bronchitis, atelectasis, birth trauma, back pain, neck pain, headache, otitis media, seizure, tetraparesis, Bell's palsy, constipation, disk herniation, lumbar fracture, hemiparesis, Osgood Schlatter, radial head subluxation, and developmental delay.

### Descriptive studies 2007 update

The 36 descriptive studies, mainly single case reports, involved a total of 138 children and reported a wide range of health conditions treated by chiropractors. The conditions include GERD, constipation, palsy, developmental delay, torticollis, elbow pain, autism, enuresis, sleep dysfunction, ADHD, wry neck, heel pain, uveitis, scoliosis, irritable baby syndrome, gastroenteritis, seizures, colic, migraine, motion sickness, otitis media and dyslexia.

### Abstracts at conference proceedings

There were 14 conference proceeding abstracts involving a total of 173 children.

Five RCT's involved asthma, autism, infantile colic (2), and otitis media with chiropractors performing all spinal manipulation. The trial on asthma was a feasibility study with 6 children. The trial on autism involved 14 children and concluded that the clinical improvement of autistic children under full spine chiropractic adjustment can be enhanced four fold and may reach to complete cure if the technique of adjustment is shifted to upper cervical. The first trial on infantile colic involved 30 children and concluded that chiropractic spinal manipulation is more effective than placebo in the treatment of the symptomatology of infantile colic. The second trial on infantile colic involved 45 children and concluded that chiropractic spinal manipulation is effective and safe in the treatment of infantile colic. The trial on chronic otitis media involved 30 children and concluded that there is a significant decrease in the number of days it takes to resolve a chronic otitis media using chiropractic care compared to one treated with antimicrobial therapy.

The nine descriptive studies involved 48 children with chiropractors performing manipulation for the following conditions: autism, headache, acute otitis media, seizure, difficult breast feeding, and torticollis.

### Abstracts at conference proceedings 2007 update

There were 17 conference proceeding abstracts involving a total of 72 children.

One observational study involving 15 children (8 in treatment group, 7 in control group) reported on the effects of joint manipulation on the performance of young swimmers.

The 16 descriptive studies involved 57 children with chiropractors performing manipulation for the following health conditions: conversion disorder, torticollis, enuresis, headache, GERD, seizure, scoliosis, ADHD, palsy and developmental delay.

## Limitations

There are several limitations in this updated review and these are consistent with our earlier review. We have relied on the databases to capture the full breadth of evidence, and while it may be comprehensive, it may not be exhaustive since some evidence may be currently in the publication process. The inclusion criteria allowed for observational and descriptive studies in addition to RCT's, and so the capture rate was high which some might argue is selection bias. Conference proceedings reported in abstract form usually lack sufficient detail to scrutinize methodology and/or data analysis, and generally have not been subjected to the same peer review process as utilized by scholarly journals. We have not addressed publication bias in child health [17]. Again, we have not treated the evidence with critical appraisal techniques because there is insufficient high quality evidence to warrant explicit statistical methodologies. In addition, the sample sizes in some of the RCT's are exceedingly small as these studies are pilot or feasibility studies. Far less weight ought to be attached to such limited trials.

## Discussion

There is a relationship between methodologic quality, quality assessment dimensions, and conclusions that may be generalizable across populations. Patients expect improving clinical outcomes, minimized risk and absolute safety. Patient health interest's are protected and advanced when health care providers increasingly rely on systematic reviews and high quality RCT's for decision making. Sound evidence-based clinical decision making is related to efficacy, effectiveness and efficiency.

Clearly, systematic reviews provide the basis to establish conclusive findings and thereby generalize these scientific findings across populations in a manner which protects and advances the health interests of patients. Our study included two systematic reviews which reached conclusions with respect to infantile colic and KISS. Our study included ten RCT's, many with very small sample sizes, but yet reaching conclusions on asthma, enuresis, infantile colic, jet lag, radial head subluxation and adolescent idiopathic scoliosis.

Health care providers, consumers and payers of health interventions require reliable information in order to determine issues such as patient-centered risks, benefits, effectiveness and safety. Health care decisions made on the basis of expert opinion or clinical experience may not be addressing these issues in the fullest context.

In our study, most evidence is clinically based at low levels on the scale of evidence and consist of 177 descriptive studies, and they are mainly single case reports. Practitioners may realize successful outcomes on a single case basis but the conclusions arrived at may be premature. Generalizing such premature conclusions to larger patient populations is a position not well grounded in science.

## Summary

There has been no substantive shift in this body of knowledge during the past 3 1/2 years. The health claims made by chiropractors with respect to the application of manipulation as a health care intervention for pediatric health conditions continue to be supported by only low levels of scientific evidence. Chiropractors continue to treat a wide variety of pediatric health conditions. The evidence rests primarily with clinical experience, descriptive case studies and very few observational and experimental studies. Interestingly, no RCT's have been published on the treatment of back pain with manipulation in a pediatric population. The health interests of pediatric patients would be advanced if far more rigorous scientific inquiry was undertaken to examine the value of manipulative therapy in the treatment of pediatric conditions. The clinical encounter needs to be better grounded with scientific evidence of much higher quality than currently exists.

## Authors' note

The reader is advised of a comprehensive systematic review that was published in June 2007 which evaluated the evidence on the effect of chiropractic care rather than spinal manipulation only, on patients with non-musculoskeletal conditions. (Hawk C, Khorsan R, Lisi A, Ferrance R, Evans M. Chiropractic care for non-musculoskeletal conditions: a systematic review with implications for whole systems research. J Alt Comp Med 2007;13(5):491–512.)

## Competing interests

The authors declare that they have no competing interests.

## Authors' contributions

AG and RR conceived this study, undertook all aspects of the work product and approved the final manuscript.

## References

[B1] Gotlib A, Rupert R (2005). Assessing the evidence for the use of chiropractic manipulation in pediatric health conditions – a systematic review. Pediatr Child Health.

[B2] Husereau D, Clifford T, Aker P, Leduc D, Mensinkai S (2003). Spinal manipulation for infantile colic.

[B3] Brand PL, Engelbert RH, Helders PJ, Offringa M (2005). Systematic review of the effects of therapy in infants with the KISS-syndrome (kinetic imbalance due to suboccipital strain). Ned Tijdschr Geneeskd.

[B4] Balon J, Aker PD, Crowther ER, Danielson C, Cox PG, O'Shaughnessy D, Walker C, Goldsmith CH, Duku E, Sears MR (1998). A comparison of active and simulated chiropractic manipulation as adjunctive treatment for childhood asthma. New Engl J Med.

[B5] Bronfort G, Evans RL, Kubic P, Filkin P (2001). Chronic pediatric asthma and chiropractic spinal manipulation: a prospective clinical series and randomized clinical pilot study. J Manip Physiol Ther.

[B6] LeBoeuf C, Brown P, Herman A, Leembruggen K, Walton D, Crisp TC (1991). Chiropractic care of children with nocturnal enuresis – a prospective outcome study. J Manipulative Physiol Ther.

[B7] Reed WR, Beavers S, Reddy SK, Kern G (1994). Chiropractic management of primary nocturnal enuresis. J Manipulative Physiol Ther.

[B8] Macias CG, Bothner J, Wiebe R (1998). A comparison of supination/flexion to hyperpronation in the reduction of radial head subluxations. Pediatrics.

[B9] Wiberg J, Nordsteen J, Nilsson N (1999). The short term effect of spinal manipulation in the treatment of infantile colic: a randomized controlled clinical trial with a blinded observer. J Manip Physio Ther.

[B10] Olafsdottir E, Forshei S, Fluge G, Markestad T (2001). Randomized controlled trial of infantile colic treated with chiropractic spinal manipulation. Arch Dis Child.

[B11] Straub WF, Spino MO, Alattar MM, Pfleger B, Downes JW, Bellizaire MA, Heinonen OJ, Vasankari T (2001). The effect of chiropractic care on jet lag of Finnish junior elite athletes. J Manipulative Physiol Ther.

[B12] Sawyer CE, Evans RL, Boline PD, Branson R, Spicer A (1999). A feasibility study of chiropractic spinal manipulation versus sham spinal manipulation for chronic otitis media with effusion in children. J Manip Physiol Ther.

[B13] Rowe DE, Feise RJ, Crowther ER, Grod JP, Menke JM, Goldsmith CH, Stoline MR, Souza TA, Kambach B (2006). Chiropractic manipulation in adolescent idiopathic scoliosis: a pilot study. Chiropractic & Osteopathy.

[B14] Brzozowske WT, Walton EV (1977). The effect of chiropractic treatment of students with learning and behavioural impairments resulting from neurological dysfunction. ACA J Chiro.

[B15] Khorshid KA, Sweat RW, Zemba DA, Zemba BN Clinical efficacy of upper cervical versus full spine chiropractic care on children with autism: a randomized clinical trial. J Vert Sublux Res.

[B16] Vallone S (2004). Chiropractic evaluation and treatment of musculoskeletal dysfunction in infants demonstrating difficulty breastfeeding. J Clin Chiropr Ped.

[B17] Klassen T, Wiebe N, Russell K, Stevens K, Hartling L, Craig W, Moher D (2002). Abstracts of randomized controlled trials presented at the Society for Pediatric Research Meeting – an example of publication bias. Arch Pediatr Adolesc Med.

